# Design and Fabrication of a Precision Template for Spine Surgery Using Selective Laser Melting (SLM)

**DOI:** 10.3390/ma9070608

**Published:** 2016-07-22

**Authors:** Di Wang, Yimeng Wang, Jianhua Wang, Changhui Song, Yongqiang Yang, Zimian Zhang, Hui Lin, Yongqiang Zhen, Suixiang Liao

**Affiliations:** 1 School of Mechanical and Automotive Engineering, South China University of Technology, Guangzhou 510640, China; scut061389@163.com (D.W.); ym_zwang@163.com (Y.W.); song_changhui@163.com (C.S.); zh_ronaldo@163.com (Z.Z.); linhui_zj@163.com (H.L.); 2 Hospital of Orthopedics, Guangzhou General Hospital of Guangzhou Military Command, Liuhua Road, Guangzhou 510010, China; yongqiang_zheng@163.com (Y.Z.); m15603000944_1@163.com (S.L.)

**Keywords:** additive manufacturing, selective laser melting, precision medical device, computer aided design, surgery template, screw insertion

## Abstract

In order to meet the clinical requirements of spine surgery, this paper proposes the fabrication of the customized template for spine surgery through computer-aided design. A 3D metal printing-selective laser melting (SLM) technique was employed to directly fabricate the 316L stainless steel template, and the metal template with tiny locating holes was used as an auxiliary tool to insert spinal screws inside the patient’s body. To guarantee accurate fabrication of the template for cervical vertebra operation, the contact face was placed upwards to improve the joint quality between the template and the cervical vertebra. The joint surface of the printed template had a roughness of Ra = 13 ± 2 μm. After abrasive blasting, the surface roughness was Ra = 7 ± 0.5 μm. The surgical metal template was bound with the 3D-printed Acrylonitrile Butadiene Styrene (ABS) plastic model. The micro-hardness values determined at the cross-sections of SLM-processed samples varied from HV0.3 250 to HV0.3 280, and the measured tensile strength was in the range of 450 MPa to 560 MPa, which showed that the template had requisite strength. Finally, the metal template was clinically used in the patient’s surgical operation, and the screws were inserted precisely as the result of using the auxiliary template. The geometrical parameters of the template hole (e.g., diameter and wall thickness) were optimized, and measures were taken to optimize the key geometrical units (e.g., hole units) in metal 3D printing. Compared to the traditional technology of screw insertion, the use of the surgical metal template enabled the screws to be inserted more easily and accurately during spinal surgery. However, the design of the high-quality template should fully take into account the clinical demands of surgeons, as well as the advice of the designing engineers and operating technicians.

## 1. Introduction

Due to developments in spinal surgery, it is necessary for many complicated surgeries (e.g., cervical spine surgery, thoracic spinal surgery, spinal deformity surgery) to fix screws into the pedicle of the vertebral arch in the spine and insert appropriate bone tissue substitutes (e.g., an inter-body fusion cage or titanium mesh) for structural reconstruction [1,2,3]. Considering the fact that the anatomical structure near the spine is very complex and has many important nerves and blood vessels, insertion of the screws has to be done precisely and carefully to ensure that no harm is done to the nerves and blood vessels [4,5].

Designing of a customized surgical template through computer aided program is emerging as a simple and feasible method [6,7,8]. In this method, images of the spine at the surgery site are collected using high-speed spiral CT before reconstructing the computer-based 3D design. Then, a navigation template model is designed using the reverse engineering software, printed on a 3D printer and is finally provided to the doctors for use. In recent years, some researchers successfully fabricated the non-metal template for inserting screws into the cervical vertebra and pedicle of vertebral arch in the thoracic vertebrae [9,10,11], resulting in improvements in precise insertion of screws. Prior to the advent of 3D metal printing, clinical medicine relies on the customized templates made from resin. Goffin, Jan, et al. [12] developed a template containing a drill guide allowing navigated screw positioning inside the left and right isthmus of C2. The rotational stability of the template toward the lamina C2 was insufficient in the first series, but for the second series both the entry points and screw trajectories were very satisfactory. Kaneyama et al. [13] studied the effectiveness of the tailor-made screw guide template (SGT) system for the placement of C2 screws, including in cases with abnormalities, the study mainly focus on the clinical usage of a non-metal template, it demonstrates that the SGT system provided extremely accurate C2 cervical screw insertion.

The resin-made template is cost-effective and easy to build, but has the following limitations [14,15]: (1) the accuracy of printing resin template is poor, as the space between the template and the metal drill may be enlarged, resulting in navigation errors; (2) the template is very brittle and, hence, it gets easily damaged during the surgical disinfection process; (3) as it is required to bind to the tissue manually, the template cannot be fixed and completely bound to the bone.

With continuous developments in Selective laser melting (SLM), the accuracy and speed of printing are improving immensely [16,17,18]. More than ten types of metal powders can be printed by SLM, including pure Ti and its alloy, CoCr alloy, stainless steel, copper, Al alloy, and tool steel, etc. The dimensional accuracy is around 50 μm and the density could reach up to 100%, and the SLM technique has been widely used in medical, aerospace, and mold industries, etc. Current research includes the densification mechanism [19], microstructure and properties [20], residual stress and its distribution [21], etc. The application of 3D metal printing in surgery is drawing more and more attention [22,23,24]. Takemoto et al. [22] developed a novel patient-specific template design that reduces the contact area without sacrificing stability; it will avoid susceptibility to intervening soft tissue, template geometric inaccuracy, and difficulty during template fitting. Their investigation not only gives more clinical cases, but also addresses more regarding the template geometric accuracy. Considering the fact that the template made via metal printing is very precise, easy to use, and disinfect, it has great potential for surgery.

In the current investigations, to meet the requirements of spinal surgery, a customized metal template for spinal surgery is fabricated in this investigation through computer aided design and 3D metal printing. The fabricated template can serve as an aid to achieve precision and customization during spinal surgery, and in improving efficacy of the treatment. This investigation focuses on the key issues pertaining to the design and metal 3D printing of a precision template for spinal surgery.

## 2. Customized Design of the Template in Spinal Surgery

The subject of the present investigations was a three-year-old patient suffering from osodontoideum and an unstable atlantoaxial joint, who needed to undergo atlantoaxial fixation surgery. The clinical operation was approved by the Hospital Ethics Committee, and the clinical usage of the 3D-printed metal template was included in the patient’s informed consent.

### 2.1. Steps for Designing the Template

The design requirements include: (1) the contact surface should completely fit the patient’s bone surface; (2) the strength of the template should be the same or better than the traditionally-made template; (3) the key positions, such as the guider hole and the locating hole, should be of sufficient accuracy.

**Figure 1:**
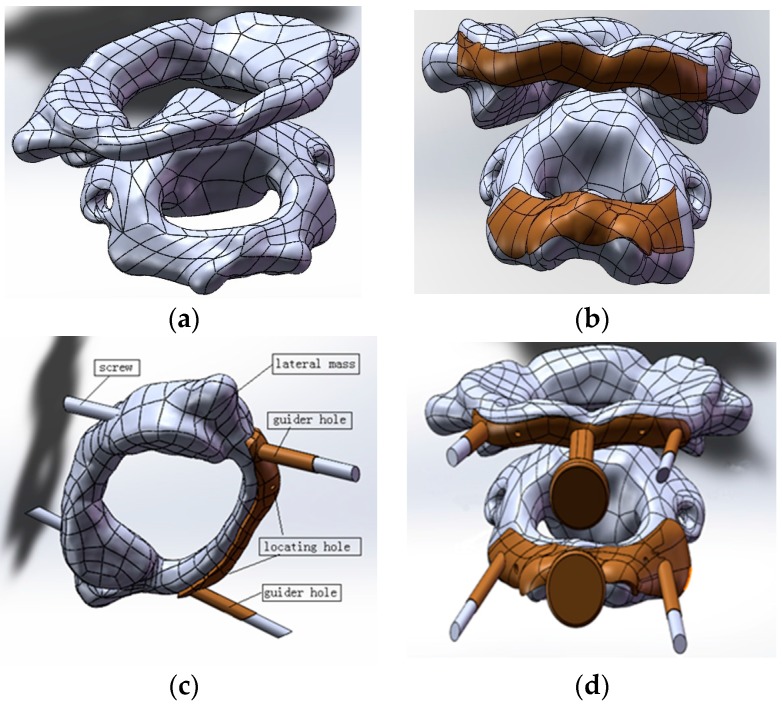
Key steps to design the spinal surgery template. (**a**) Atlas (up) and axis (down); (**b**) curved surface and cervical vertebra; (**c**) guider hole and locating hole; and (**d**) template with the handle and cervical vertebra.

- **Step 1:** Extraction and processing of the target spine model [2,3]
  The Biograph 64 PET/CT apparatus (SIEMENS, Munich, Germany) was used to obtain scan data, with a scan condition of 120 kv,150 mAs; scan slice thickness of 0.5 mm; and a scanning time of 10–15 s. The factor that affects the geometrical accuracy is the scan slice thickness, which would impact the surface fitting accuracy. The data from a thin-layer CT scan of the cervical vertebrae of the patient was imported into Mimics 16.0 (Materialise, Leuven, Belgium) to extract the target spine. Store this as STL (binary) to be imported into Geomagic software (3D syestems, Rock Hill, SC, USA). Elimination of the noise points inside the bone to obtain the 3D entity was accomplished by executing the following process: extract surface—construct patches—construct grids—surface fitting, and store it in the STEP AP203 format; import the data from Geomagic software into SolidWorks software; and integrate the separate 3D entity models into an assembled model to obtain the target spine model (as shown in Figure 1a).
- **Step 2:** Design of the contact surface of the template in the spinal surgery
  In the atlantoaxial fixation surgery, the curved surface is chosen to be at the arcus posterior of the cervical vertebra. A rectangle of appropriate size is clipped and then the curved surface is thickened outwardly (as shown in Figure 1b).
- **Step 3:** Choice of the locations of the guider hole and locating hole
  The criterion for choosing the guider hole is to enable the nail to reach the lateral mass and to minimize the surface of the arcus posterior atlantis. The screws should be buried in the lateral mass to bypass important nerves and blood vessels. The locating hole should be placed at a distance of around 10 mm from the center of the template, with its direction normal to its curved surface (as shown in Figure 1c).
- **Step 4:** Design of the handle
  In order to stabilize the surgical template while inserting the screws, a handle is placed in the middle of the template for the doctor to hold. The long column is clamped with the forefinger and the disk is pressed with the thumb. Edges and corners of the handle are chamfered to make the clamping comfortable. The final proposed design of the template is shown in Figure 1d.

### 2.2. Geometrical Parameters of the Surgical Template

Geometrical parameters of the spinal surgery template as shown in Table 1, the criteria behind the values assigned to the exposed geometrical parameters are detailed as follow:

(a) The template’s thickness was designed to be 0.8–1.0 mm: (1) this value is assigned to guarantee the template’s strength and hardness, so as to resist deformation or fracture during the clinical operation; (2) the thinner the template is, the better the contact between the template and the bone surface should be. Based on above two reasons, the template’s thickness is designed to be 0.8–1 mm.
(b) The guider hole diameter was designed to be 2.7 mm: this dimension is designed according to the screw’s size.
(c) The guide’s wall thickness was designed to be 0.15 mm: as long as the strength is ok, the guider hole’s wall thickness should be as thin as possible
(d) The guide’s length was designed to be 10 mm: as long as the screw placement is stable, the guide’s length should be as short as possible.
(e) The locating hole size was designed to be 1.0 mm: this dimension is designed according to the screw’s size.
(f) The locating holes’ distance to the center was designed to be 10 mm, (1) in order to secure the template to the bone surface through tiny screws during clinical operation; and (2) the locating holes should be placed as close to the guider holes as possible, so as to guarantee stability during the screw placement.

## 3. Experimental Techniques and Procedures

### 3.1. Equipment, Materials, and Optimization for the SLM Process

**Figure 2:**
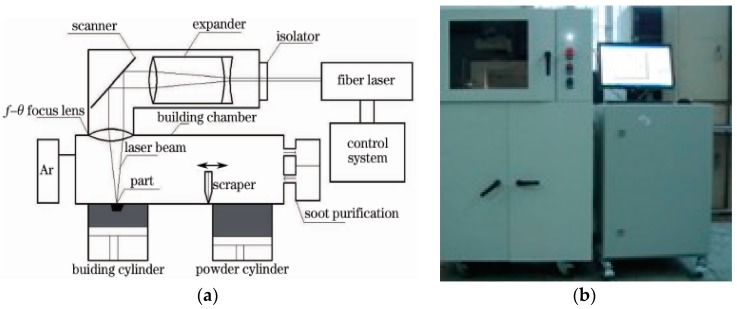
Principle of SLM manufacturing and the experimental DiMetal-100 equipment. (**a**) Principle of SLM manufacturing; and (**b**) DiMetal-100.

The experiments were carried out using a self-developed selective laser melting (SLM) machine, DiMetal-100 (SCUT, Guangzhou, China). Figure 2a shows the principle of SLM manufacturing and the DiMetal-100 equipment used is shown in Figure 2b. The setup consisted of a fiber laser, optical-path transmission unit, sealing chamber (including the powder recoating device), mechanical drive, and controlling systems, as well as the processing software. The laser was directed using a scanning galvanometer, which was then focused through the f-θ lens, and melts the metallic powders on the plane selectively, followed by stacking them layer-wise into metal parts. The machine has a scanning speed in the range between 10–5000 mm/s, thickness of the processing layer in the range 20–100 µm, and a laser focusing spot diameter of 70 µm. The largest size of the part produced was 100 mm × 100 mm × 120 mm. Since the powder was fully melted during the process, protection of the SLM-processed parts from oxidation was essential. Therefore, processing of all of the metal powders were carried out in an argon or nitrogen atmosphere, with not more than 0.15% O_2_.

**Figure 3:**
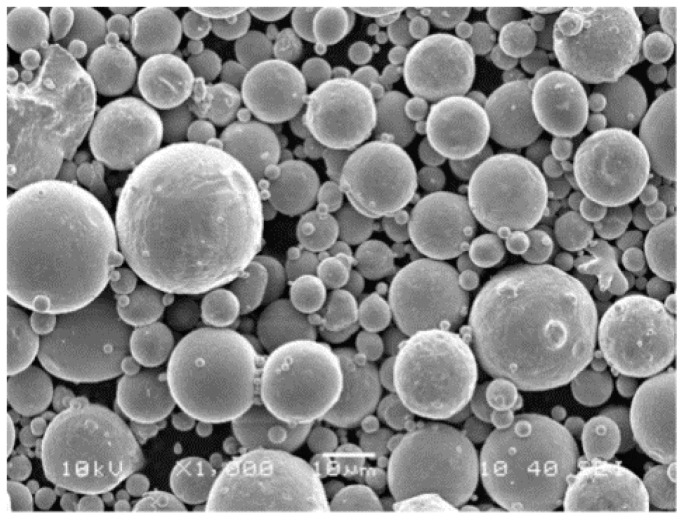
The SEM image of 316L stainless steel powder having the size of 500 mesh.

The material used in this current investigation was gas-atomized 316L stainless steel provided by LPW Technology Ltd. from Manchester, UK, with an average particle size of about 17 μm, and an apparent density of the powder of about 4.04 g/cm^3^. Figure 3 shows the scanning electron microscopy (SEM) image of 316L stainless steel powder having the size of 500 mesh.

The optimized process was obtained through multiple technological optimizations. First, the laser spot diameter was set to be unchangeable, and orthogonal experiments were designed to determine the laser power, scanning speed, layer thickness, scanning spacing, and the target is to obtain dense and high-surface-quality parts. Then, further experiments were designed to determine the exact laser energy input by changing the laser power and scanning speed. the optimized SLM fabricating parameters of the stainless steel powder are shown in Table 2.

**Figure 4:**
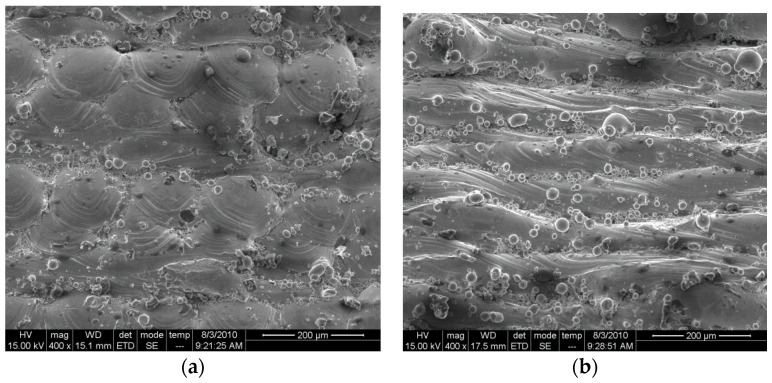
Microscopic analysis of surface morphology of stainless steel parts from SLM. (**a**) Surface morphology of the side part; and (**b**) surface morphology of the upper part.

Figure 4a,b shows the microscopic views of the morphologies of the side and upper surfaces, respectively, from which the compact overlapping effect between adjacent tracks and layers could be seen. Some finely-unmelted powdered particles can be seen, which remained adhered to the junctions of the layers and tracks. The similar phenomenon has been reported in the literature, according to which this occurred mainly due to the laser heat effect [25]. Figure 4b shows that the melting tract at the upper surface of the cube appeared like ripples, and some unmelted particles were also found adhered to the surface.

### 3.2. Design-Manufacturing Procedures of Template

**Figure 5:**
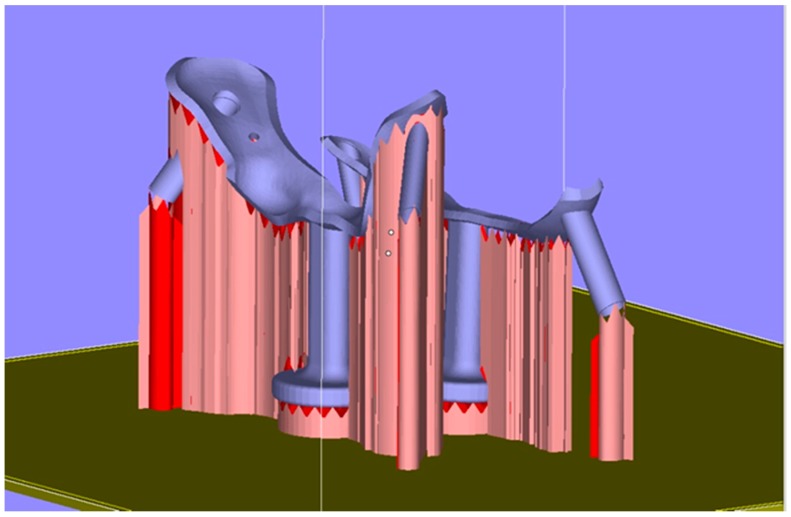
Location adjustment and addition of supports for the surgical template model.

1. A customized spinal surgery template was designed under the guidance of a surgeon (Hospital of Orthopedics, Guangzhou General Hospital of Guangzhou Military Command).
2. The designed surgical template model was exported as a STL data file and was imported into Magics 16.0 to adjust the location of the model (Figure 5). The data on triangular slices was restored, the metal supports were added and the other necessary operations were carried out. The contact face between the template and the vertebra was placed upwards to ensure high surface quality.
3. The slicing operations were carried out by setting the parameters for laser spot compensation and layer thickness in Magics 16.0 (Materialise, Leuven, Belgium), and then the data for laser scanning track control was generated.
4. The processing data was input into the self-developed 3D metal printer, DiMetal-100, to build the template, and then the precision and mechanical properties of the fabricated surgical template were evaluated.
5. Finally, the spinal surgery template was put into clinical use to evaluate its efficacy, and the key technologies for fabrication of the precision spinal surgery template were evaluated.

### 3.3. Testing Procedures

The JB-8c type contact stylus roughness meter (Guangjing, Ningbo, China) was used for measuring the roughness value Ra. The JB-8c roughness meter has a maximum measurement value of 640 μm, a measurement accuracy of 0.4 μm, a measurement resolution of 0.01 μm, and a measurement rReproducibility of 2%. The sampling length was 0.8 mm, evaluation length was 4 mm, and measurement speed was 0.32 mm/s. The hardness was tested using a digital micro-hardness HVS-1000 apparatus (Shunhua, Shenzhen, China). The measurement resolution of the HVS-1000 was 0.01 μm, with a measurement range of 1~3000 HV. The tensile strength of the manufactured part using SLM was tested using an electronic universal testing machine CMT5105 (MTS, Eden Prairie, MN, USA). The CMT5105 has a relative error value of the test force of ±0.5%, the resolution of the test force was 1/300,000 FS, the relative error value of the deformation was ±0.50%, and the displacement resolution was 1 μm.

## 4. Results and Discussion

### 4.1. Results of SLM Manufacturing for the Spinal Surgery Template

**Figure 6:**
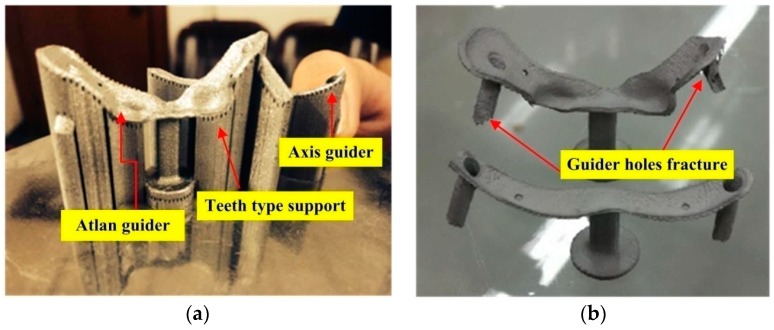
Spinal surgery template obtained from SLM. (**a**) Surgical template without removing the support; and (**b**) surgical template after abrasive blasting.

The real template entity as obtained through SLM is shown in Figure 6a. Then, the metal supports were removed followed by sand blasting and thus resulting the final structure as shown in Figure 6b.

From Figure 6, it can be seen that the surgical template was well printed, without any obvious deformations. It was further observed that the two guider holes in the axis surgery template were broken while manually removing the support. T can be explained by the fact that the wall of the two holes in the axis template was as thin as 110 μm, whereas the laser spot was only 70 μm. Considering the effect of laser irradiation, the melting channel was usually in the range 100–120 μm after melting and solidification of the laser radiated powder. Hence, when the scanning interval between the melting channels was 80 μm, the wall of the template hole was as thick as compared to the width of the melting channel and the support. Hence, the metal support was easily broken during manual removal when it was placed under the guider hole.

### 4.2. Manufacturing Accuracy and Mechanical Property

#### 4.2.1. Manufacturing Accuracy

**Figure 7:**
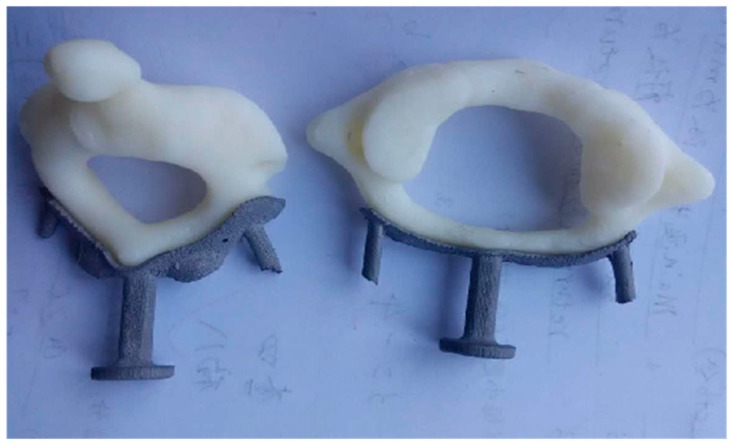
Fitting of the metal template and the FDM-printed cervical vertebra model.

The cervical vertebra model was printed with ABS using the FDM uPrintSE Plus printer from Stratasys. (Eden Prairie, MN, USA). The joint between the template and the ABS model was inspected as shown in Figure 7. Through measurements by vernier caliper, the SLM fabrication accuracy of the template in the current study was 0.1 ± 0.05 mm, and the FDM fabrication of the ABS spine models was around 0.2 ± 0.05 mm, the gap dimension was measured around 0.2 ± 0.05 mm. The above dimensions fully qualify it as a metal template.

The most important part of the template was the contact face for clinical applications. After measuring the surface roughness of the contact face between the template and the ABS model, it was found that the roughness value of the contact face in the metal template changed from Ra = 13 ± 2 μm to Ra = 7 ± 0.5 μm after the shot blast. The surface roughness of the template after the shot blast was much higher than that of the traditionally fabricated parts. These are serious defects that limit the widespread acceptance of 3D metal printing in many industrial areas. However, for clinical applications, a rough surface helps in close binding of the metal template and the site of interest in the patient’s body.

**Figure 8:**
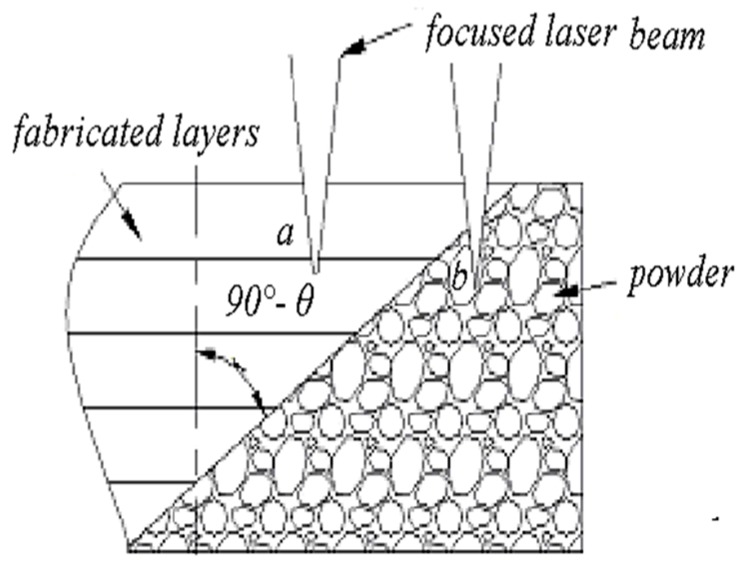
Illustration of laser scanning the overhang.

Visual observation showed that the lower surface of the template was rougher than that of the upper surface. Thus, it can be inferred that, during data processing, the contact face between the template and the cervical vertebra should be placed upwards to guarantee surface quality of the contact face. Figure 8 illustrates the fact that the upper surface of the metal 3D printed part was higher than the lower surface. As shown in Figure 8, the laser spot wholly penetrated into the powder-supported area (point b). Given the same laser processing parameters, the energy input of the powder supported area was much larger than that of the solid material supported zone, because the thermal conductivity of the powder zone was only 1/100 of that of the corresponding solid material [25]. In this case, the melting pool in the powder support area was very large and sink into the powder due to the action of gravity and capillary force, which resulted in poor quality of the downward surface.

#### 4.2.2. Hardness and Mechanical Property Measurement

As per the available literature, it has been widely accepted by academia that the metal 3D-printed parts outperform the traditionally-cast parts in terms of hardness and tensile strength [26]. These are approximately equal to, or even surpass, the mechanical properties of the wrought parts, but possessing somewhat low extensibility [27]. In this experiments, the micro-hardness measured at the cross-sections of SLM-processed samples varied from HV_0.3_ 250 to HV_0.3_ 280, the high value owing to the high densification level and refined microstructures. The tensile strength measured was in the range of 450 MPa to 560 MPa, according to the ASTM standard at 515 MPa, which was also comparable to those of the wrought 316L stainless steel. The elongation was measured from 7.5% to 8.6%, which was a little lower than the ASTM standard due to the inevitable metallurgical defects within the SLM parts.

### 4.3. Clinical Test of the Customized Spinal Surgery Template

**Figure 9:**
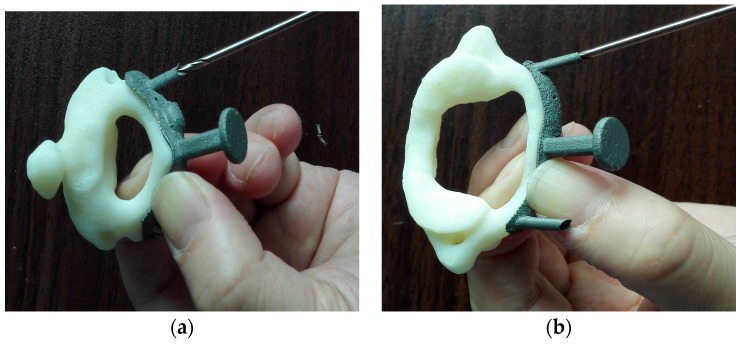
In vitro test of the metal templates. (**a**) Axis template; (**b**) atlas template.

After sand blasting, the metal template was subjected to high-pressure disinfection before clinical experimentation; the close-up pictures of the templates during the in vitro model test are shown in Figure 9.

**Figure 10:**
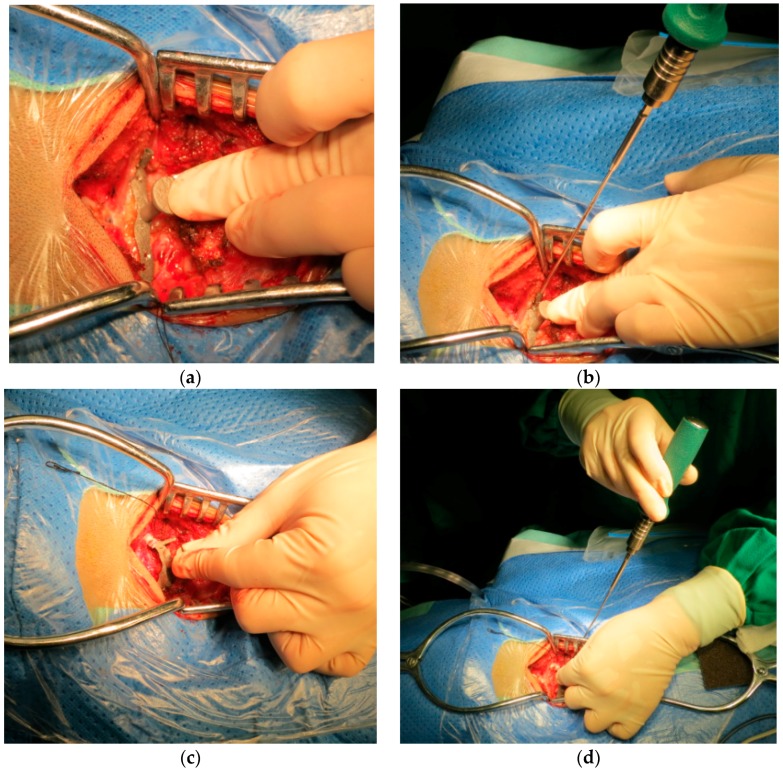
(**a**) Use of the atlas template; (**b**) insertion of screws into atlas using the template; (**c**) use of the axis template; (**d**) insertion of screws into the axis during the operation involving the insertion of high-precision screws with templates.

The whole surgery lasted about two and a half hours, and desired results were achieved in the clinical operation, as shown in Figure 10, which illustrated the operation of insertion of the high-precision screws with atlas and axis templates. The authors confirmed the direction of the drill by C-arm as the intraoperative navigation, and the C-arm is also used to confirm whether the screws insert in the desired trajectory.

While fabricating the template using 3D metal printing, the precision in navigation was improved in the following ways: (1) the length of the template hole was increased; a special drill was used to facilitate the insertion of screws, and the need to change the path of the screws during insertion was eliminated. The length of the guider hole was set to 10 mm in the present investigation to alleviate instability caused by the gap during surgery; (2) several locating holes were added in the template and small screws were provided so that the template could be fixed closely to the surface of the bone of interest with the screws. A locating hole with a diameter of 1 mm was set at the left and right sides of the template respectively to further improve the drilling accuracy.

**Figure 11:**
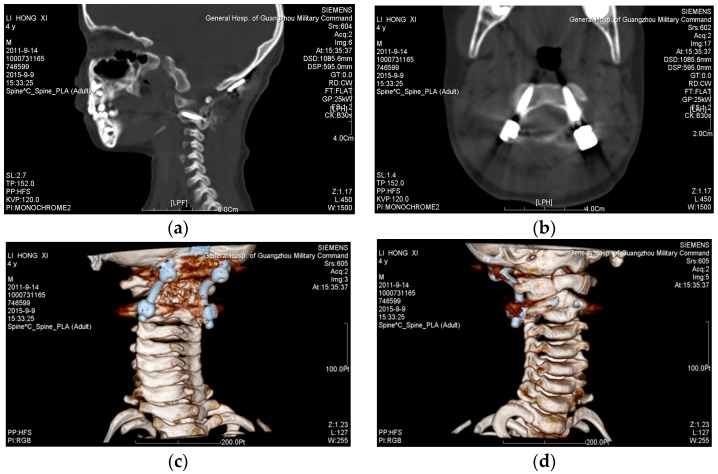
The surgical results observed through the CT reverse engineering method. (**a**) Sagittal view; (**b**) transverse view; (**c**) 3D orientation 1; and (**d**) 3D orientation 2.

Figure 11 shows the surgical results observed through the CT reverse engineering method; it can be seen that the screws were precisely placed in the desired location after the surgical operation.

### 4.4. Discussion

**Figure 12:**
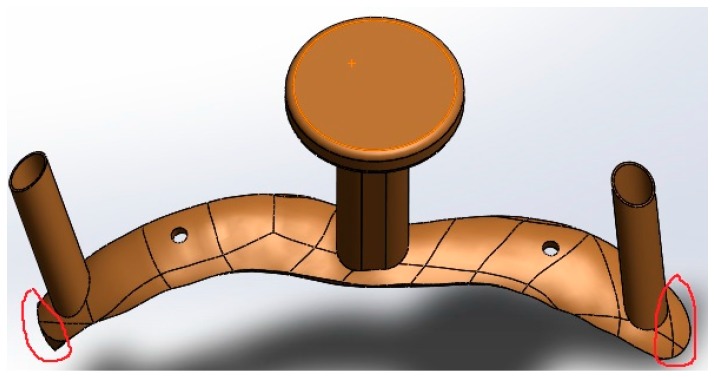
Redundant parts of the atlas template (marked in red).

Compared with the existing resin template, the metal template manufactured in the current investigation have high navigation accuracy, could be easily subjected to autoclaving, and were found robust against damage and deformation during the operation. However, the design and 3D metal printing of the metal spinal surgery template in these experiments are required to be improved in the following ways:

(1) A locating hole with a diameter of 2.3 mm was recommended for the template. It was set to 1 mm in the current study. A small size of the locating hole made it difficult for a naked eye to spot the hole. Adjusting the diameter of the locating hole according to industrial standards on the screws justified the use of standard screws.
(2) The two key screw holes were required to enlarge their diameters to 3.0 mm. The diameters of these screw holes were set to 2.7 mm. The reason why the sizes of the parts were smaller than the design was that some powder particles remained adhered to the wall of the holes, due to laser radiation. Detailed analysis on the principles is given below.
(3) The thickness of the walls of the screw holes was increased to 0.3 mm to prevent walls of the hole from breaking during the removal of the support by sand blasting. As discussed in Section 3.1, the strength of the hole could be guaranteed by setting the width of the melting track to 100–120 μm, scanning interval to 80 μm, wall thickness of the hole to 0.3 mm or the stacking thickness of about four melting tracks.
(4) The curved surface in current experiment was so wide that the incision made in the surgery was very large, which caused more harm to the patient. Hence, for the design of the customized spinal surgery template, redundant parts should be removed. Taking the atlas template in Figure 12 as an example, the parts circled red in the designed model should be eliminated.

**Figure 13:**
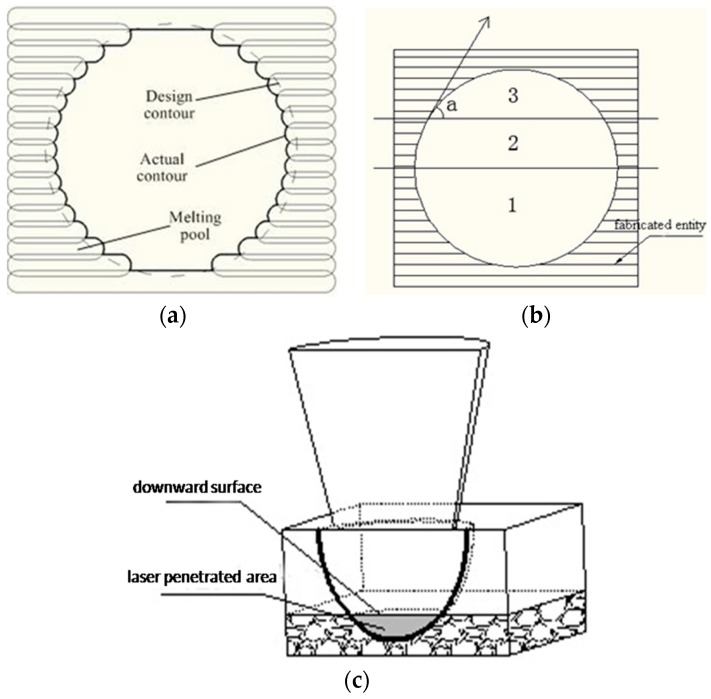
Limitations of principles for fabricating two classes of round holes. (**a**) The contour error of round holes parallel to the Z axis; (**b**) the three stages in fabricating an overhang hole; and (**c**) an illustration of deep laser penetration.

Improvements should also be made in the support and the round holes of the metal 3D-printed spinal templates. as per the following suggestions:

1. The criterion for adding the support is to ensure that the support can be easily removed after the forming process and it remains stable and free from warpage. At the same time, heat conduction should be guaranteed during the forming process in order to reduce accumulation of thermal stress in the template. The added supports in the current investigation were very dense with high strength, which made it difficult to remove the supports, leaving the guider holes prone to fracture. The further optimization of the customized metal template should focus on the parameters pertaining to the teeth between the endpoints of the support and the template, including the gap between the teeth, penetration of teeth into the parts, and teeth width.
2. The techniques for fabricating the screw holes should be improved. When the spatial locations of parts are adjusted in Magics 16.0, the locations of holes in the template should also change. Based on the way in which the holes incline, the 3D metal printed holes are categorized into three classes: round holes parallel to the fabricating direction, round holes vertical to the fabricating direction, and round holes making an angle with the X-Y plane. In what follows, limitations of principles for fabricating the first two classes of round holes will be discussed as depicted in Figure 13.

**Figure 14:**
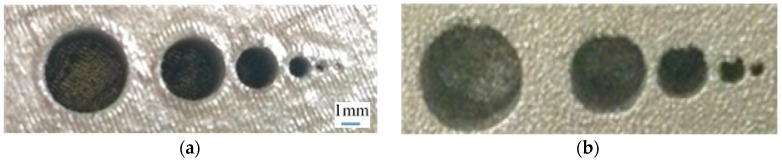
Small round holes fabrication experiments. (**a**) Fabricated round holes parallel to the Z axis; (**b**) Fabricated round holes vertical to the Z axis.

Round holes parallel to the fabricating direction: the dimensional error of the round holes parallel to the fabrication direction is mainly a result of the contour error [28]. Figure 13a shows the scanning line filling the round hole. The designed contour is round (shown by dotted lines in Figure 13a). Due to the width of the melting track, the actually fabricated contour is represented by the bold black line in the Figure 13a. Powder adhesion also affects dimensional accuracy of round holes. The largest diameter of the powder used in this paper is 53 µm. Powder adhesions to the interior walls of the round holes cause a reduction in the diameter of the holes, as shown in Figure 14a. In addition to the scanning strategy, thermal cycles in SLM can also cause dimensional inaccuracy of 316L [21].

Round holes vertical to the fabricating direction: considering the fabrication of overhanging round holes (as shown in Figure 13b), this can be divided into three stages. In the first stage, there is no overhanging structure, so this section of round holes can be fabricated stably. In the second stage, the round holes can still be fabricated smoothly, as the inclined angle а is sufficiently large, so the extent of overhanging between adjacent layers is less, and the laser penetration (Figure 13c) is not obvious. In the third stage, the small inclined angle a, coupled with large amount of overhanging leads to great laser penetration and “adherent dross” [29]. When the size of the hole decreases to be small enough, the “adherent dross” will even block the holes, as Figure 14b shows.

Based on the above analysis it can be summarized that the holes fabricated by SLM have dimensional errors, which is a principle error and cannot be avoided. However, several measures can be adopted to obtain the desired holes, such as enlarging the holes at the design stage and using traditional drilling methods in the post-processing stage. The latter method was used in the current study. That is, the holes were enlarged using the drill after the metal template was fabricated, to ensure that the diameter of locating holes matched with the standard screws. The key point was to ensure that the sizes of the left and right guider holes were suited for the screws to be inserted. Generally, the diameter of guider hole has to be slightly larger than the inserted screw so that the product has better navigation accuracy and is easier to use.

As the user of the template, the surgeons have the final decision on the design and optimization of the template. However, usually the surgeons lack expertise in the customization of complex curved surfaces. In addition to this, an experienced designer must tailor geometrical sizes of the parts as per the needs of the surgeons, such as the height of the template, location and size of the locating holes, and location and size of the guider holes. Finally, the designers should take into account the limitations of principles in 3D metal printing. For example, the surface roughness of the 3D metal printed parts is inferior to the traditional method. The vertical hole fabricated through 3D metal printing has adherent dross or blockage. The resolution of the thin parts and tiny cylinders fabricated through 3D metal printing cannot be less than the width of a single melting track. Therefore, fabricating a high-precision metal template that is suitable for clinical application requires synergy between doctors, designers, and 3D printing engineers. Compared to the traditional technology of screw insertion, the use of the surgical metal guider enabled the screws to be inserted more easily and accurately during the spinal surgery. By using the method proposed in this study, the patients with similar symptoms could adopt this accurate template. The main difference among patients is the customized template design (the doctor is critical to the design), as each patient has a unique contact surface. Aside from this, the other procedures should be the same.

The future enhancement of the spine templates need the optimization of each stage from design to clinical operation, The screw’s placement result (position and direction) is the key target in this study, and the screw’s placement result is closely related to template’s design, SLM fabrication process, and the post-process. As the anatomical landmarks are often not easily defined, the trajectory of the screws are also not very easily decided, and the doctor’s clinical experience is always very important for the trajectory design. Amirouche et al. [30] developed a method and mathematical lemmas for generating automated optimal safe screw insertion trajectories based on the identification of a set of intrinsic parameters. The template’s design requires the communication of the clinical doctor, designer, and SLM process engineer for the design of the patient-specific template, which is the key to this technique, and can be used widely.

## 5. Conclusions

The surgical guide is customized in the present study to the needs of the patients through 3D metal printing. In addition to perfectly matching the dissection requirements of patients, it can insert the screws accurately and avoid iatrogenic complications. Moreover, it does not rely on the experience of surgeons, or expensive navigation devices and fluoroscopy during operation. The following conclusions can be drawn:

1. A customized surgical template with tiny locating holes was successfully fabricated through computer-aided design and selective laser melting to assist the doctors in inserting high-precision screws into the spine. The design innovation in this study is that tiny locating screw holes were added on the template, the two locating holes are used for making the template contact the bone surface firmly by using two tiny screws, without needing to press the metal template by hand during clinical operation. The proposed metal template provided high navigation precision, was robust against damage, and could be easily autoclaved.
2. To ensure accurate fabrication of the spinal surgery template, the contact face was placed upwards, which resulted in better surface quality of the contact face between the template and the spine. The roughness of the contact face was Ra = 13 ± 2 micrometers for the printed template and Ra = 7 ± 0.5 micrometers after abrasive blasting. The metal template was bound with the ABS model. The micro-hardness measured at the cross-sections of SLM-processed samples varied from HV_0.3_ 250 to HV_0.3_ 280, and the measured tensile strengths were in the range of 450–560 MPa.
3. Improvements in the design of the template and 3D metal printing are required. For example, the wall thickness of the two guider holes in the axis guide should be increased to avoid fracturing. The size of the locating holes and guider holes should also be enlarged. Redundant parts of the model should be removed to prevent the excessively wide, curved surface from causing more pain to the patients.

## Figures and Tables

**Table 1:** Geometric parameters of the spinal surgery template.

Guider Position	Thickness (mm)	Left Guider Hole			Right Guider Hole			Left Locating Hole		Right Locating Hole	
		Inner Diameter(mm)	Wall Thickness(mm)	Length	Inner Diameter(mm)	Wall Thickness (mm)	Length(mm)	Inner Diameter(mm)	Distance to the Center (mm)	Inner Diameter (mm)	Distance to the Center (mm)
Atlas guider	0.8	2.7	0.15	10.0	2.7	0.15	10.0	1.0	12.45	1.0	10.84
Axis guider	1.0	2.7	0.11	10.0	2.7	0.11	10.0	1.0	11.08	1.0	10.72

**Table 2:** The optimized SLM fabricating parameters.

Laser Power (W)	120
Scanning Speed (mm/s)	600
Scanning Space (µm)	80
Layer Thickness (µm)	25

## References

[1] Kabins M.B., Weinstein J.N. (1991). The history of vertebral screw and pedicle screw fixation. Iowa Orthop. J..

[2] Manbachi A., Cobbold R.S., Ginsberg H.J. (2014). Guiderd pedicle screw insertion: Techniques and training. Spine J..

[3] Ma T., Xu Y.Q., Cheng Y.B., Jiang M.Y., Xu X.M., Xie L., Lu S. (2012). A novel computer-Assisted drill guider template for thoracic pedicle screw placement: A cadaveric study. Arch. Orthop. Trauma Surg..

[4] Eftekhar B., Ghodsi M., Ketabchi E., Rasaee S. (2002). Surgical simulation software for insertion of pedicle screws. Neurosurgery.

[5] Ryken T.C., Kim J., Owen B.D., Christensen G.E., Reinhardt J.M. (2009). Engineering patient-specific drill templates and bioabsorbable posterior cervical plates: A feasibility study. J. Neurosurg. Spine.

[6] Lund T., Laine T., Österman H., Yrjönen T., Schlenzka D. (2012). Accuracy of computer assisted pedicle screw insertion: The evidence. J. Bone Jt. Surg. Br..

[7] Ryken T.C., Owen B.D., Christensen G.E., Reinhardt J.M. (2009). Image-based drill templates for cervical pedicle screw placement. J. Neurosurg. Spine.

[8] Melkent T., Foley K.T., Estes B.T., Chaudoin J. (2002). Image Guided Spinal Surgery Guide, System, and Method for Use Thereof. U.S. Patent.

[9] Owen B.D., Christensen G.E., Reinhardt J.M., Ryken T.C. (2007). Rapid prototype patient-specific drill template for cervical pedicle screw placement. Comput. Aided Surg..

[10] Xu N., Wei F., Liu X., Jiang L., Cai H., Li Z., Yu M., Wu F., Liu Z. (2016). Reconstruction of the upper cervical spine using a personalized 3D-printed vertebral body in an adolescent with ewingsarcoma. Spine.

[11] Fu M., Lin L., Kong X., Zhao W., Tang L., Li J., Ouyang J. (2013). Construction and accuracy assessment of patient-specific biocompatible drill template for cervical anterior transpedicular screw (ATPS) insertion: An in vitro study. PLoS ONE.

[12] Goffin J., Van Brussel K., Martens K., Vander Sloten J., Van Audekercke R., Smet M.H. (2001). Three-dimensional computed tomography-based, personalized drill guide for posterior cervical stabilization at C1–C2. Spine.

[13] Kaneyama S., Sugawara T., Sumi M., Higashiyama N., Takabatake M., Mizoi K. (2014). A novel screw guiding method with a screw guide template system for posterior C-2 fixation: Clinical article. J. Neurosurg. Spine.

[14] Sarment D P., Sukovic P., Clinthorne N. (2003). Accuracy of implant placement with a stereolithographic surgical guide. Int. J. Oral Maxillofac. Implant..

[15] Di Giacomo G.A., Cury P.R., de Araujo N.S., Sendyk W.R., Sendyk C.L. (2005). Clinical application of stereolithographic surgical guides for implant placement: Preliminary results. J. Periodontol..

[16] Bremen S., Meiners W., Diatlov A. (2012). Selective laser melting. Laser Tech. J..

[17] Leuders S., Thöne M., Riemer A., Niendorf T., Tröster T., Richard H.A., Maier H.J. (2013). On the mechanical behaviour of titanium alloy TiAl6V4 manufactured by selective laser melting: Fatigue resistance and crack growth performance. Int. J. Fatigue.

[18] Hasan R., Mines R.A.W. Variations in diameter of struts for micro-lattice structure manufactured using selective laser melting. Proceedings of the Mechanical Engineering Research Day 2016, Centre for Advanced Research on Energy.

[19] Wu W., Tor S.B., Chua C.K., Leonga K.F., Merchant A. (2015). Investigation on processing of ASTM A131 Eh36 high tensile strength steel using selective laser melting. Virtual Phys. Prototyp..

[20] Lam L.P., Zhang D.Q., Liu Z.H., Chua C.K. (2015). Phase analysis and microstructure characterisation of AlSi10Mg parts produced by Selective Laser Melting. Virtual Phys. Prototyp..

[21] Yadroitsev I., Yadroitsava I. (2015). Evaluation of residual stress in stainless steel 316L and Ti6Al4V samples produced by selective laser melting. Virtual Phys. Prototyp..

[22] Takemoto M., Fujibayashi S., Ota E., Otsuki B., Kimura H., Sakamoto T., Kawai T., Futami T., Sasaki K., Matsushita T. (2016). Additive-manufactured patient-specific titanium templates for thoracic pedicle screw placement: Novel design with reduced contact area. Eur. Spine J..

[23] Verweij J.P., Moin D.A., Mensink G., Nijkamp P., Wismeijer D., van Merkesteyn J.P. (2016). Autotransplantation of Premolars With a 3-Dimensional Printed Titanium Replica of the Donor Tooth Functioning as a Surgical Guide: Proof of Concept. J. Oral Maxillofac. Surg..

[24] Cornelius C.P., Smolka W., Giessler G.A., Wilde F., Probst F.A. (2015). Patient-specific reconstruction plates are the missing link in computer-assisted mandibular reconstruction: A showcase for technical description. J. Maxillofac. Surg..

[25] Foroozmehr A., Badrossamay M., Foroozmehr E. (2016). Finite element simulation of selective laser melting process considering optical penetration depth of laser in powder bed. Mater. Des..

[26] Attar H., Prashanth K.G., Chaubey A.K., Calin M., Zhang L.C., Scudino S., Eckert J. (2015). Comparison of wear properties of commercially pure titanium prepared by selective laser melting and casting processes. Mater. Lett..

[27] Kasperovich G., Hausmann J. (2015). Improvement of fatigue resistance and ductility of TiAl6V4 processed by selective laser melting. J. Mater. Process. Technol..

[28] Salonitis K. (2016). Design for additive manufacturing based on the axiomatic design method. Int. J. Adv. Manufac. Technol..

[29] Wang D., Yang Y.Q., Liu R.C., Xiao D.M., Sun J.F. (2013). Study on the designing rules and processability of porous structure based on selective laser melting (SLM). J. Mater. Process. Technol..

[30] Amirouche F., Solitro G.F. (2016). Innovative Approach in the Development of Computer Assisted Algorithm for Spine Pedicle. Med. Eng. Phys..

